# αB-Crystallin Peptide Fused with Elastin-like Polypeptide: Intracellular Activity in Retinal Pigment Epithelial Cells Challenged with Oxidative Stress †

**DOI:** 10.3390/antiox12101817

**Published:** 2023-09-30

**Authors:** Sara Aly Attia, Anh Tan Truong, Alvin Phan, Shin-Jae Lee, Manal Abanmai, Marinella Markanovic, Hugo Avila, Haozhong Luo, Atham Ali, Parameswaran G. Sreekumar, Ram Kannan, J. Andrew MacKay

**Affiliations:** 1 Department of Pharmacology and Pharmaceutical Sciences, Mann School of Pharmacy and Pharmaceutical Sciences, University of Southern California, Los Angeles, CA 90089, USA; saraatti@usc.edu (S.A.A.); truonga@alumni.usc.edu (A.T.T.); alvinpha@usc.edu (A.P.); abanmai@usc.edu (M.A.); marinella.markanovic@gmail.com (M.M.); hugoavila08@gmail.com (H.A.); haozhong@usc.edu (H.L.); athamali@usc.edu (A.A.); 2 Mann Department of Biomedical Engineering, Viterbi School of Engineering, University of Southern California, Los Angeles, CA 90089, USA; shinjae@usc.edu; 3 Department of Pharmaceutics, College of Pharmacy, Prince Sattam bin Abdulaziz University, Al Kharj 11942, Saudi Arabia; 4 Doheny Eye Institute, Pasadena, CA 91103, USA; sparameswaran@doheny.org (P.G.S.); rkannan@doheny.org (R.K.); 5 Stein Eye Institute, Geffen School of Medicine, University of California, Los Angeles, CA 90095, USA; 6 Department of Ophthalmology, Keck School of Medicine, University of Southern California, Los Angeles, CA 90089, USA

**Keywords:** oxidative stress, crystallin, peptide, transition temperature ‘T_t_’, DLS, 3D culture, lysosome, confocal microscopy, immunofluorescence

## Abstract

Background: Oxidative stress-induced retinal degeneration is among the main contributing factors of serious ocular pathologies that can lead to irreversible blindness. αB-crystallin (cry) is an abundant component of the visual pathway in the vitreous humor, which modulates protein and cellular homeostasis. Within this protein exists a 20 amino acid fragment (mini-cry) with both chaperone and antiapoptotic activity. This study fuses this mini-cry peptide to two temperature-sensitive elastin-like polypeptides (ELP) with the goal of prolonging its activity in the retina. Methods: The biophysical properties and chaperone activity of cry-ELPs were confirmed by mass spectrometry, cloud-point determination, and dynamic light scattering ’DLS’. For the first time, this work compares a simpler ELP architecture, cry-V96, with a previously reported ELP diblock copolymer, cry-SI. Their relative mechanisms of cellular uptake and antiapoptotic potential were tested using retinal pigment epithelial cells (ARPE-19). Oxidative stress was induced with H_2_O_2_ and comparative internalization of both cry-ELPs was made using 2D and 3D culture models. We also explored the role of lysosomal membrane permeabilization by confocal microscopy. Results: The results indicated successful ELP fusion, cellular association with both 2D and 3D cultures, which were enhanced by oxidative stress. Both constructs suppressed apoptotic signaling (cleaved caspase-3); however, cry-V96 exhibited greater lysosomal escape. Conclusions: ELP architecture is a critical factor to optimize delivery of therapeutic peptides, such as the anti-apoptotic mini-cry peptide; furthermore, the protection of mini-cry via ELPs is enhanced by lysosomal membrane permeabilization.

## 1. Introduction

Oxidative stress-induced retinal degeneration is implicated in many degenerative pathologies [1,2,3]. Age-related macular degeneration (AMD) is such a condition, which affects 12.6% of Americans according to the Vision and Eye Health Surveillance System [4]. A healthy retina is normally exposed to environmental oxidative stressors such as air pollution [5], light [6], or chronic conditions like diabetes mellitus or associated lung diseases [7,8,9]. These promote reactive oxygen species (ROS), which accumulate and activate apoptotic signaling [10]. Homeostasis of the retina requires an equilibrium between ROS production and neuroprotective mechanisms that mitigate the effects of ROS [11]. When their dysregulation reaches pathological levels, such conditions require interventions to restore balance and protect vision [12,13]. Combating retinal dysfunction by administrating long-lasting, neuroprotective therapies is one possible solution [14,15,16]. As members of the small heat shock protein (sHsps) family, α-crystallin proteins are naturally secreted chaperones that protect against stress through multiple biological mechanisms. These include interfering with protein denaturation, regulating proteasome degradation, halting inflammatory responses, and blocking apoptotic signaling [17,18,19]. It was shown by Hinton and coworkers [20] that α-crystallin knockout mice are more susceptible to oxidative stress-induced cellular apoptosis. Later, Wang and coworkers [21] reported that loss of either αA or αB-crystallins increased retinal inflammation and decreased photoreceptor survival in the P23H Autosomal Dominant Retinitis Pigmentosa mouse model. α-crystallins mainly consist of A and B classes, which are characterized by two β-sheets of 6–8 strands and truncated by N and C termini that are modulated by post-translational modifications [22,23]. For example, Smulders and coworkers [24] proposed that immobilization of the C-terminus of bovine αA-crystallin reduced protein thermostability, and therefore its functional integrity. Both crystallins are prominent in ocular tissues, including the retinal pigment epithelium (RPE), lens, ganglion cell layers, and photoreceptors, as well as in other body tissues [18,20]. Multiple groups have explored the chaperone activity of α-crystallin-derived fragments, compared to the parent protein [25,26]. Sharma and coworkers [26,27] identified a minimal 19 amino acid ‘mini-cry’ peptide (DFVIFLDVKHFSPEDLTVK) from αA-crystallin, which blocked aggregation of mutant αAG98R protein when observed using chromatographic and spectrometric methods. αAG98 is also a mutant form of αA-crystallin, at which this mutation was found to be associated with autosomal dominant cataract. Interestingly, this short peptide alone was capable of stabilizing αAG98R protein, thus maintaining its biological function. In another study, Bhattacharyya and coworkers [25] demonstrated that the 73–92 residues of αB-crystallin, encoded DRFSVNLDVKHFSPEELKVK, display an integral substrate binding site, 83–90 region, in addition to their anti-aggregation function. Despite these impressive experimental activities, the therapeutic potential of the mini-cry peptide in vivo likely requires controlled release to extend the duration of effect. To do so, drug delivery strategies may increase its local mean residence time, and thus reduce the necessity for frequent administration. Provided such a method maintains chaperone and biological signaling activity in the retina, it may be possible to harness these and related peptides as therapeutics.

Our group uses one such platform for controlled release that leverages ELPs. These are based on pentapeptide sequences, (VPGXG)_n_, by which ‘n’ specifies the molecular weight and the guest residue ‘X’ determines the polymeric solubility. To provide a high molecular weight platform to display mini-cry, ELP fusions can be engineered through recombinant DNA methodology. As smart biopolymers, ELPs have tunable thermo-responsive properties. Below a solution T_t_, ELPs are highly soluble, remain optically clear, and easily pass through a narrow-gauge needle. Upon injection to the body and once above T_t_, the ELPs coacervate into micron-sized droplets. Coacervation hinders local clearance and extends the period of absorption at the point of administration. Selection of ELP length and hydrophobicity fundamentally modify this phase separation behavior, thus affecting the rate of dissolution and duration of therapeutic protection [28,29,30]. The conjugation of peptides to ELP allows their thermo-responsive deposition, which can be engineered to retain cargo and enhance therapeutic outcomes.

Previously, our team reported the engineering of αB-crystallin mini-peptide to an ELP SI encoded as follows: (VPGSG)_48_(VPGIG)_48_ [14]. This ELP is composed of an amphiphilic diblock copolymer with hydrophilic and hydrophobic guest residues, which promotes formation of supramolecular micelles. Due to its diblock composition, it exhibits two T_t_; furthermore, this formulation was efficacious against an induced model of geographic atrophy of the retina [31]. For the first time in this paper, our group fused this mini-cry peptide to an ELP monoblock called V96 motif: (VPGVG)_96_. Compared to SI, V96 uses only valine as a hydrophobic guest residue. While the fusion of SI to mini-cry decreased the bulk phase transition temperature, T_t2,_ from around 77 to 30 °C, both V96 and cry-V96 phase separate near 37 °C. With the goal of designing a simpler mini-cry ELP for future pharmacokinetic and therapeutic studies, this study compares their physiochemical properties, anti-apoptotic signaling, and cellular uptake.

## 2. Materials and Methods

### 2.1. ELPs Cloning, Expression, Production, and Concentration Assessment

ELP genes were constructed in a pET25b (+) vector by recursive directional ligation as previously explained [32]. In brief, sense and antisense DNA oligonucleotides encoding the αB-crystallin peptide sequence, GDRFSVNLDVKHFSPEELKVKG, were chemically synthesized (Genewiz, South Plainfield, NJ, USA) 5′ phosphorylated complementary oligonucleotides; forward sequence:

5′TATGGGTGATAGATTTAGCGTTAACCTGGACGTGAAACATTTCTCCCCAGAAGAACTGAAAGTGAAGGGTTATTAGACTCCTCG3′ and reverse sequence:

5′ATCCGAGGAGTCTAATAACCCTTCACTTTCAGTTCTTCTGGGGAGAAATGTTTCACGTCCAGGTTAACGCTAAATCTATCACCCA3′.

Forward and reverse 5′ phosphorylated complementary oligonucleotides were annealed in water at 95 °C for 5 min and cooled gradually to room temperature for 1 h. These were ligated (T4 DNA ligase, New England BioLabs, Ipswich, MA, USA #M0202S) into a pET25b (+) vector double digested with NdeI and BamHI-HF and agarose gel purified (New England BioLabs, Ipswich, MA, USA #R0111S and #R3136S). The ligation mixture was transformed into TOP10 cells (Thermo Fisher Scientific, Waltham, MA, USA #C404010) and selected on LB agar supplemented with carbenicillin (100 μg/mL carbenicillin) (GoldBio, Olivette, MO, USA #C-103-100). Colonies were expanded and plasmids were extracted by QIAprep^®^ miniprep kit (Qiagen, Germantown, MD, USA #27106). For confirming insertion of the mini-cry gene into the vector, diagnostic DNA digests were reviewed using gel electrophoresis and confirmed by Sanger sequencing with T7 specific primers (Genewiz, South Plainfield, NJ, USA). To link the mini-cry sequence to a gene encoding V96, two vectors were ligated: one encoding an ELP and one encoding just the mini-cry peptide. The V96 vector contains a single BseRI restriction cut site just after its start codon. The mini-cry vector contains a single BseRI restriction cut site just downstream of the mini-cry gene. Thus, both plasmids were double digested with BssHII and BseRI (New England BioLabs, Ipswich, MA, USA #R0199S and #R0581S), and the correct fragments were gel purified, ligated, and plasmid DNA was screened by Sanger sequencing. For expression, electrocompetent ClearColi BL21 (DE3, BioCat GmbH, Lucigen, WI, USA #60810-1) cells were transformed by electroporation. The transformed cells were spread on LB agar plate with carbenicillin and incubated at 37 °C. A single bacterial colony was inoculated into 50 mL of autoclaved LB Broth Miller media (Millipore Sigma, St. Louis, MO, USA #L3522). Following the overnight incubation at 37 °C in an orbital shaker, the starter culture was transferred to 6 L of LB media until the optical density at 600 nm (OD_600_) reached between 0.6 and 0.8, which is optimal for protein yield. Expression was induced by 500 μM of isopropyl β-D-1-thiogalactopyranoside (IPTG) (GoldBio, Olivette, MO, USA #I2481C50) and incubated at room temperature. The following day, the bacterial cells were pelleted, resuspended in cold phosphate-buffered saline (PBS), and disrupted by probe-tip sonication for 3 min (10 s on, 20 s off) on ice. The lysate was treated with 0.5% polyethylenimine (PEI) (Millipore Sigma, St. Louis, MO, USA #408700) and centrifuged 10,000× RPM for 15 min at 4 °C to remove insoluble debris. The clarified lysate was then heated in a water bath at 37 °C and powdered sodium chloride (NaCl) was added to a concentration of 2.5 M until coacervation was observed. This solution was centrifuged for 15 min at 37 °C (4000 RPM) to collect the ELP in the pellet. The supernatant was discarded and the pellet was resolubilized in cold PBS. This ELP-mediated purification cycle was repeated 3–4 rounds, while decreasing the amount of NaCl used. To ensure sterility and reduce endotoxin, ELPs were filtered using Acrodisc^®^ Mustang E Membrane filters (Pall Life Sciences, Port Washington, NY, USA #MSTG25E3). To further reduce endotoxin, immobilized polymyxin B chromatography was used (Abcam, Cambridge, MA, USA, #ab239707). The final endotoxin level was confirmed by the chromogenic LimulusAmebocyteLysate (LAL, Lonza, Walkersville, MD, USA #N294-06) assay. For determining the concentration, ELPs were mixed with 6 M guanidine chloride (1:5 ratio) (Thermo Fisher Scientific, Waltham, MA, USA #24115) and a nanodrop spectrophotometer measured absorbance at 280 nm and 350 nm (Thermo Fisher Scientific, Waltham, MA, USA). Beer–Lambert’s law was employed for concentration calculation as follows:

(1) $$C=\frac{A280-A350}{\varepsilon l}$$

At which C (M) is the ELP concentration, ε (M^−1^ cm^−1^) is the molar extinction, and l (cm) is the path length. As reported previously, the extinction coefficient value was estimated as 1285 M^−1^ cm^−1^ [14]. 

### 2.2. ELP Purity

The purity of ELPs was evaluated by sodium dodecyl sulfate−polyacrylamide gel electrophoresis (SDS−PAGE). A total of 10 μg per 15 μL of ELPs were mixed with β-mercaptoethanol in Laemmli buffer (Thermo Fisher Scientific, Waltham, MA, USA #31350010 and Bio-Rad, Hercules, CA, USA #1610747, respectively) at a 1:4 ratio, denatured at 95 °C for 5 min, loaded on a 4−20% gradient Mini-Protean TGX precast gel (Bio-Rad, Hercules, CA, USA #456-1095), and run for 32 min at 200 volts. The gel was stained by G-250 Coomassie (Bio-Rad, Hercules, CA, USA #1610786) and imaged by ChemiDoc Touch Image System (Bio-Rad laboratories, CA, USA).

### 2.3. Exact Mass Determination

ELP mass was confirmed by Matrix-Assisted Laser Desorption/Ionization Mass spectrometry (MALDI-TOF MS, Bruker, Billerica, MA, USA). A total of 5 μg of ELPs were diluted into 2,6 dihydroxyacetophenone (DHAP, Thermo Fisher Scientific, Waltham, MA, USA #114810250) solution (10 mg/mL DHAP into 50% acetonitrile) (Acros Organics, Morris Plains, NJ, USA #AC114820100) and supplemented by 0.1% formic acid (Thermo Fisher Scientific, Waltham, MA, USA #28905). Less than 1 μL of this solution was spotted on a 384-Big Anchor MALDI target and air-dried until crystallization. Crystallized samples were further analyzed by the raplifleX MALDI-TOF system and plotted by GraphPad Prism software, version 9.4.0.

### 2.4. Rhodamine Labeling

NHS-rhodamine (Thermo Fisher Scientific, Waltham, MA, USA #46406) was dissolved in dimethyl sulfoxide (DMSO) to 10 mg/mL. It was mixed slowly at 1:3 a molar ratio with cry-SI and cry-V96. The reaction was stirred overnight at 4 °C. To remove the unbound dye, the mixture was dialyzed across a 10,000 MWCO membrane against sterile 1× phosphate-buffered saline (PBS) for at least 4 changes over 48 h. Following dialysis, samples were sterilized by 0.2 μm Supor^®^ membrane (Pall Life Sciences, Port Washington, NY, USA #4612). The concentration of labeled rhodamine (C) was calculated using a DU800 UV/vis spectrophotometer (Beckman, Brea, CA, USA) at OD_555_ nm using the following equation:

(2) $$C_{rhodamine}=\frac{A555}{\varepsilon l}$$

where ε = 70,000 M^−1^ cm^−1^, l = 0.1 cm, and A555 is the absorbance at 555 nm. The labeling efficiency was determined by the following:

(3) $$E=\frac{C_{rhodamine}}{C_{ELP}}\times 100\%$$

Purified samples were assessed by SDS-PAGE on a 20% gradient Mini-Protean TGXprecast gel and rhodamine fluorescence was detected by the iBright FL1000 system according to the selected wavelength from (Thermo Fisher Scientific, MA, USA).

### 2.5. Transition Temperature

To quantify the ELP phase behavior, ELPs were diluted in PBS to multiple concentrations and loaded into quartz microcuvettes. The optical density was recorded by DU800 UV/vis spectrophotometer at 350 nm. The temperature (~11 to 85 °C) was heated at 1 °C/min and the T_t_ was defined at the point of maximum slope.

(4) $$T_{t}=b-mLog\left[C_{ELP}\right]$$

where a 10× change in concentration will yield a change of m °C, and b is the *y*-axis intercept for a T_t_ of 1 μM ELP. Subsequently, ELP coacervates were visualized at 25 and 37 °C using phase contrast microscopy. An amount of 25 μM of ELPs were plated at 35 mm glass-bottom culture dishes, placed into a heating chamber, and imaged by Keyence microscope (Keyence, Itasca, IL, USA) using a 40× lens.

### 2.6. Dynamic Light Scattering

To evaluate hydrodynamic radius, R_h_, of ELPs, proteins were assessed under different temperatures (DynaPro Plate 2.0 Reader, Wyatt Instruments, Santa Barbra, CA, USA). Prior to analysis, 25 μM ELP samples at 4 °C were filtered through a Whatman Anotop^®^ 0.02 μm filter (Millipore Sigma, St. Louis, MO, USA #WHA68091002) to remove residual precipitates. A total of 60 μL samples were plated in triplicate into a 384-well black plate with a clear bottom (GreinerOne, Monroe, NC, USA #82051-294), covered by 15 μL mineral oil (Ward’s Science, Rochester, NY, USA #470301-505) to avoid evaporation, and centrifuged (1000× RPM) to remove air bubbles. The data were analyzed by Dynamics V7 software.

### 2.7. Chaperone Activity Assay

For assessing the chaperone activity of the four ELPs, 0.4 mg/mL insulin from bovine pancreas (Millipore sigma, St. Louis, MO, USA #I6634-50MG) was monitored by DLS as indicated above. Insulin aggregation was triggered upon supplementation with 80 mM Dithiothreitol (DTT) (Millipore Sigma, Burlington, MA, USA #10197777001) at a 0.7:1 molar ratio of insulin to peptide in 60 μL volume. DTT-induced aggregation of insulin and the activity of ELP and mini-cry peptides were monitored at 10 °C. This low temperature was selected to avoid coacervation phase behavior of ELPs at higher temperatures. Data were used to compare the mass distribution of ELPs particles alone and with insulin in the presence of DTT.

### 2.8. Cell Culture of ARPE-19 under Hydrogen Peroxide Challenge Using TUNEL, Viability, and Immunofluorescence Assays

To select the proper dose of hydrogen peroxide (H_2_O_2_) for screening studies, ARPE-19 cells were obtained from (American Type Culture Collection (ATCC), Manassas, VA, USA #CRL-2302), cultured in T-75 cm^2^ flasks, incubated under 37 °C and 5% CO_2_ in DMEM/F-12 medium (ATCC, Manassas, VA, USA #30-2006), supplemented with 10% fetal bovine serum (FBS) (Corning^®^, Corning, NY, USA #35-011-CV), and media was replenished every 48 h. At 70–80% confluency, cells were detached by 0.05% (*w*/*v*) Trypsin-EDTA (Thermo Fisher Scientific, Waltham, MA, USA #25300054), seeded on fresh flasks, incubated overnight, starved in medium with only 1% FBS, and treated for 24 h with different concentrations of H_2_O_2_ (Millipore Sigma, St. Louis, MO, USA #H1009). The terminal deoxynucleotidyl transferase dUTP nick end labeling (TUNEL, Millipore Sigma, St. Louis, MO, USA #11684795910) assay was used to visualize apoptotic cells according to manufacturer instructions. Images were obtained at 10× using a Keyence fluorescence microscope (Supplementary Figure S1). Over similar concentrations of H_2_O_2_, cell viability was monitored using a formazan assay. An amount of 5 × 10^3^ of ARPE-19 cells were seeded in a 96-well clear bottom plate (Genesee Scientific, El Cajon, CA, USA #25-109, starved in 1% FBS, and incubated with H_2_O_2_ doses for 24 h. Viability was detected by the colorimetric WST-1 reagent (Millipore Sigma, St. Louis, MO, USA #11644807001) (Supplementary Figure S2). For apoptosis assessment, immunofluorescence was used to visualize cleaved caspase-3. Briefly, ARPE-19 cells were seeded in Poly-D-Lysine-coated black/clear microplates (Thermo Fisher Scientific, Waltham, MA, USA #152037), starved for one day, treated with 25 μM of ELPs for one day, challenged with H_2_O_2_ for one day, and fixed with 3% paraformaldehyde (Thermo Fisher Scientific, Waltham, MA, USA #43368-9M) in PBS for 15 min. Cells were permeabilized with 0.1× Triton (Millipore Sigma, St. Louis, MO, USA #T8787) in PBS for 20 min, stained with by 4′,6-diamidino-2-phenylindole (DAPI, Thermo Fisher Scientific, Waltham, MA, USA #D1306) for 5 min, blocked by 1% BSA (Millipore Sigma, St. Louis, MO, USA #A9647-50G) in PBS for one hour, and incubated with (1:60) anti-cleaved caspase-3 antibody (Cell signaling, Danvers, MA, USA #9661) overnight at 4 °C. Apoptosis was then stained using Alexa Fluor anti-rabbit 488-conjugated secondary antibody (Thermo Fisher Scientific, Waltham, MA, USA #A-21206) at a 1:250 dilution for one hour at room temperature. Then, the treatment conditions were imaged at 10× with a Keyence fluorescence digital microscope.

### 2.9. Intracellular Localization of cry-ELPs in ARPE-19 Cells under the H_2_O_2_ Challenge

For tracking translocation under H_2_O_2_ challenge, 5 ×10^3^ of ARPE-19 cells were seeded in a 96-well plate, 1% FBS-starved, pre-treated with 25 μM ELPs for one day, and then exposed to 25 μM H_2_O_2_. Following the fixation and permeabilization, ELPs were detected using the (1:60) anti-ELP AK-1 (Cancer Therapeutics Laboratories, Los Angeles, CA, USA) antibody [33] as detected by Alexa Fluor 488-conjugated anti-mouse secondary antibodies at 1:250 (Thermo Fisher Scientific, Waltham, MA, USA #A-11001). Nuclei were stained with DAPI. Cells were imaged 20× using Airy Scan super-resolution confocal laser scanning microscopy (Zeiss, Thornwood, NJ, USA).

### 2.10. 3D Spheroid Culture

A total of 15 × 10^3^ of ARPE-19 cells were seeded in Corning^®^ spheroid microplates (Millipore sigma, St. Louis, MO, USA #CLS4515), centrifuged at 1000× RPM for 5 min immediately after seeding, incubated at 37 °C and 5% CO_2_, and supplemented by extra media every alternate day until the spheroids matured in diameter (~200 µm) at day 5. As indicated above of 2D intracellular localization study, the same treatment steps were applied.

### 2.11. Lysosomal Trafficking of ELPs

For evaluating ELP fate following cellular uptake, 5 × 10^3^ of ARPE-19 cells were seeded in a 96-well plate, incubated overnight, starved in 1% FBS for one day, co-treated with 25 μM of Rhodamine-labelled ELPs and 200 μM of H_2_O_2_ for one hour, fixed, permeabilized, blocked, incubated with Lysosome-Associated Membrane Protein-1 (LAMP-1, Cell Signaling, Danvers, MA, USA #9091S) at 1:70 dilution ratio overnight at 4 °C, washed three times with PBS for 15 min, stained with (1:250) Alexa Fluor 488-conjugated anti-rabbit secondary antibody (Thermo Fisher Scientific, Waltham, MA, USA #A-21206), nuclei were counterstained by Hoechst, and treatment conditions were visualized by confocal fluorescence microscopy. This colocalization study is open label. Data were collected from three different biological triplicates and the statistical colocalization was evaluated across all pixels of field of view Pearson’s Correlation Coefficient ‘PCC’ using ImageJ software, version 1.5.4F.

### 2.12. ELP and Dextran Uptake Assays

Following the intracellular trafficking of ELPs and dextran, cells were treated under the previously stated conditions. Following treatment, cells were incubated overnight with 25 μM of 70 kDa of anionic, fixable FITC-Dextran (Thermo Fisher Scientific, Waltham, MA, USA #D1822). Cells were fixed with 3% paraformaldehyde, stained by Hoechst (Thermo Fisher Scientific, Waltham, MA, USA #62249), and imaged by Zeiss confocal laser scanning microscopy. The laser band-pass and neutral-density filters, confocal pinhole/aperture, master gain voltage, and digital gain settings were optimized separately for each study, but were held consistent between panes shown in the same figure. Dextran/ELPs colocalization study is open label. Data were collected from three different biological triplicates and the statistical colocalization was evaluated by PCC across all pixels of field of view using ImageJ software, version 1.5.4F.

### 2.13. Statistical Methods

The colocalization analysis was processed by ImageJ ‘JACoP plugin’ software, 1.5.4F (National Institutes of Health, Bethesda, MD, USA). All data were collected at least in triplicate and statistics were analyzed by Student’s *t* test or One-Way Analysis of Variance ‘ANOVA’ followed by Tukey’s multiple comparison test using GraphPad Prism software, version 9.4.0 (San Diego, CA, USA) or IBM SPSS Statistics software, version 29.0.1.0(171) (IBM Corp, Armonk, NY, USA). To meet the homogeneity of variance assumption, a logarithmic transformation was used before performing statistical comparison for the insulin chaperone activity assay.

## 3. Results

### 3.1. Purification and Characterization of ELP Constructs

**Figure 1:**
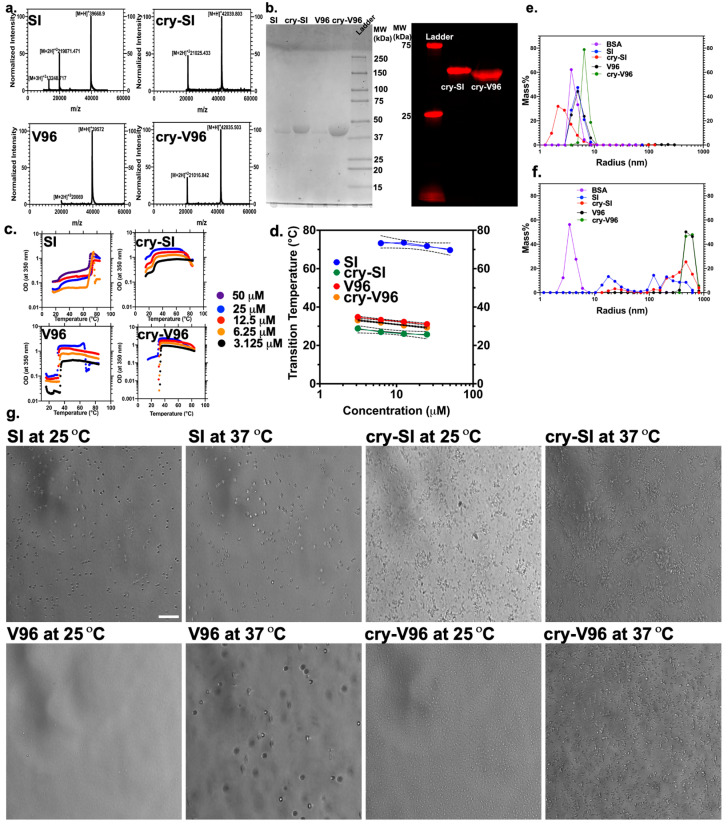
Physiochemical characterization reveals that ELP crystallin conjugation with ‘mini-cry’ promotes phase separation at physiological temperatures. (**a**) ELP constructs exhibited the expected mass as evaluated by Matrix-assisted laser desorption ion mass spectrometry. (**b**) The relative MW and purity of ELPs were confirmed by SDS-PAGE stained by Coomassie blue for non-labeled (left) and rhodamine-labeled ELPs (right). (**c**) The T_t_ characteristics of ELP assembly were measured by optical density at 350 nm as a function of temperature. Based on the maximum first derivative, the T_t_ showed a log-linear correlation with concentration. (**d**) There is an inverse relationship ‘Equation (4)’ between the phase transition temperature and concentration (mean slope ± 95% confidence interval). Using DLS, ELPs formed a monodisperse population below the T_t_ at (**e**) 10 °C, compared to (**f**) 37 °C. Below T_t_ at 10 °C, all ELPs showed one mass population with R_h_ < 10 nm. (**g**) Phase contrast microscopy also revealed noticeable differences in coacervate structures of different ELPs at 37 °C, compared to 25 °C. Scale bar = 50 µm.

SI, cry-SI, V96, and cry-V96 (Table 1) were cloned into a pET25b (+) vector, expressed in ClearColi^TM^ bacteria, and purified by ELP-mediated phase separation to produce pure protein. All peptides exhibited yields of 50–60 mg/L bacterial cultures. The relative mass and purity of ELPs were confirmed by MALDI-TOF MS (Figure 1a) and G250-Coomassie SDS-PAGE (Figure 1b). MALDI-TOF showed that ELPs and conjugates had a spectrum of at least singly and doubly charged peaks within less than 1.5% deviation from the molecular mass estimated from the open reading frame. SDS-PAGE revealed a single band of all constructs at the expected position. Following confirming the ELPs purity, their temperature-concentration phase behavior was estimated using optical density (Figure 1c), DLS analysis (Figure 1e,f), and microscopy (Figure 1g). We investigated T_t_ at higher concentrations for SI than the other peptides to observe its high bulk phase separation (Figure 1c). As shown (Figure 1d), there is an inverse relationship between the concentration and temperature by UV-vis spectrophotometry at OD_350_. This method was used to determine T_t_ as a function of concentrations. At 25 μM, SI exhibited two T_t_ at 71.8 (bulk transition temperature) and 25.8 °C (critical micelle temperature ‘CMT’) because of the presence of hydrophilic and hydrophobic guest residues, serine and isoleucine, respectively. Interestingly, the conjugation of cry peptide to SI led to a notable decrease in these two T_t_ to 25.7 and 17.7 °C, respectively. On the other hand, 25 μM of V96 and cry-V96 demonstrated T_t_ around 30 °C, which is consistent with the behavior of valine ELPs. These results indicate a noticeable reduction in the T_t_ of cry-SI, compared to SI within all observed concentrations. That might be due to interaction between the mini-cry peptide and the diblock copolymer backbone of ELPs. On the contrary, there is little difference in T_t_ between V96 and cry-V96, which suggests the cry-V96 behaves more predictably than cry-SI. Next, DLS was used to assess the R_h_ of polypeptides at 10 and 37 °C. As represented by (Figure 1e), below the T_t_ at 10 °C, all ELPs showed one mass population below the 10 nm range. This uniform, small size is consistent with soluble ELPs below their T_t_. Above T_t_ (Figure 1f), SI and cry-SI assembled into two larger populations around 20 and 400–500 nm which is consistent with their diblock architecture. The SI diblock is known to assemble ~20 R_h_ micelles; furthermore, addition of mini-cry promotes a larger population of micro-scale particles [14,34]. In contrast, above their T_t_, V96 and cry-V96 show one micron-scale population with an R_h_ ~ 600 nm. This is consistent with the monoblock construction of V96. Assembly of larger droplets of coacervate is commonly observed above the T_t_ for ELPs. Due to the large size of these droplets, phase contrast microscopy above T_t_ was used to evaluate their morphology at 37 and 25 °C (Figure 1g). The microscopy setup used to collect these images was unable to operate at more extreme temperatures, which may be required to visualize differences between ELPs above and below T_t_. As observed, SI droplets look similar at 25 and 37 °C: 25 °C is close to CMT, while 37 °C slightly exceeds the CMT (25.8 °C); however, only V96 shows a large difference between the two temperatures. This is consistent with a better defined, more predictable phase separation of V96. While not as clear as for V96, cry-V96 also has a dramatic change in droplet morphology upon heating to 37 °C. While cry-V96 assembles more rounded, distinct particles at 37 °C, cry-SI demonstrates more interconnected particles. Overall, these are consistent with DLS measurements that suggest the cry-V96 and cry-SI droplets may differ in their association into drug delivery depots.

### 3.2. Chaperone Activity of cry-ELPs

**Figure 2:**
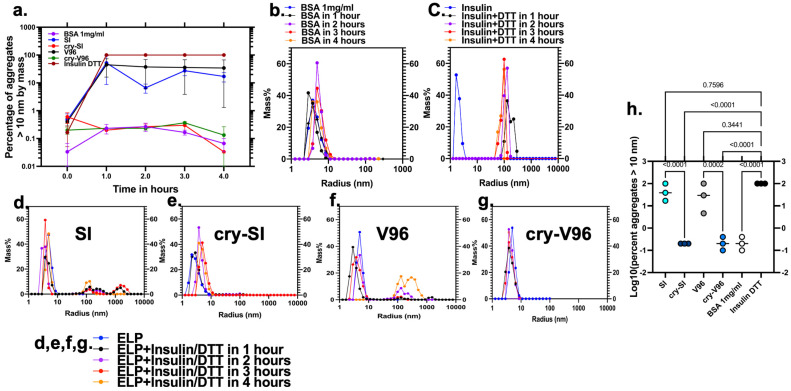
cry-ELPs exhibit chaperone activity as monitored by light scattering. (**a**) An insulin/DTT chaperone assay was monitored using DLS (*n* = 3, mean standard deviation ‘SD’) as a percentage of mass with R_h_ > 10 nm over time. (**b**) BSA without DTT (negative control) retained the R_h_ of a stable protein. (**c**) Insulin with DTT (positive control) aggregates completely into large particles. (**d**,**f**) SI and V96 were unable to halt the aggregation of insulin. (**e**,**g**) cry-SI and cry-V96 blocked aggregation of insulin. (**h**) The percent of mass in >10 nm aggregates were compared only at the one-hour timepoint because the other timepoints did not represent independent studies. ANOVA identified significant differences between groups, from which significant post hoc comparisons are indicated along with corresponding *p*-values. cry-SI and cry-V96 blocked insulin aggregation, while SI and V96 did not.

Chaperone proteins block protein misfolding, and even the short mini-cry peptide displays this activity [14]. Thus, V96 and SI with mini-cry were evaluated for relative chaperone activity against insulin denaturation induced by DTT. When insulin misfolds, its aggregates scatter light (Figure 2a); therefore, DLS was used to monitor insulin misfolding as a percentage of mass within aggregates > 10 nm over time (Figure 2a). DLS has been widely applied in chaperone activity-related studies [35,36,37,38]. As a negative control, the size of BSA was confirmed to remain constant over time (Figure 2b). In contrast, insulin starts with the R_h_ of a small protein. Upon addition of DTT, insulin disulfide bonds are broken [39], and the peptides aggregate into particles greater than 10 nm (Figure 2c). Insulin alone converts all the detectable mass into aggregates. Since ELP light scattering is temperature-dependent, the insulin chaperone assay was performed at 10 °C, below the T_t_ of the ELPs (Figure 1d). When insulin is mixed with plain ELP at a 0.7 to 1 molar ratio (70 µM of insulin to 100 µM ELPs), mass distribution increases upon addition of DTT (Figure 2d,f); however, since SI and V96 do not contain cysteine and do not aggregate at 10 °C, their mass remains in a population below 10 nm. Thus, when mixed with SI and V96, insulin aggregates do not usually reach 100% of the sample. In sharp contrast, insulin aggregation is completely blocked by cry-SI and cry-V96 (Figure 2e,g). Only ELPs displaying the mini-cry peptide prevent insulin aggregation, and no significant difference between cry-SI and cry-V96 was detected (Figure 2h).

### 3.3. Exogenous cry-ELPs Protect ARPE-19 Cells against Oxidative Stress

**Figure 3:**
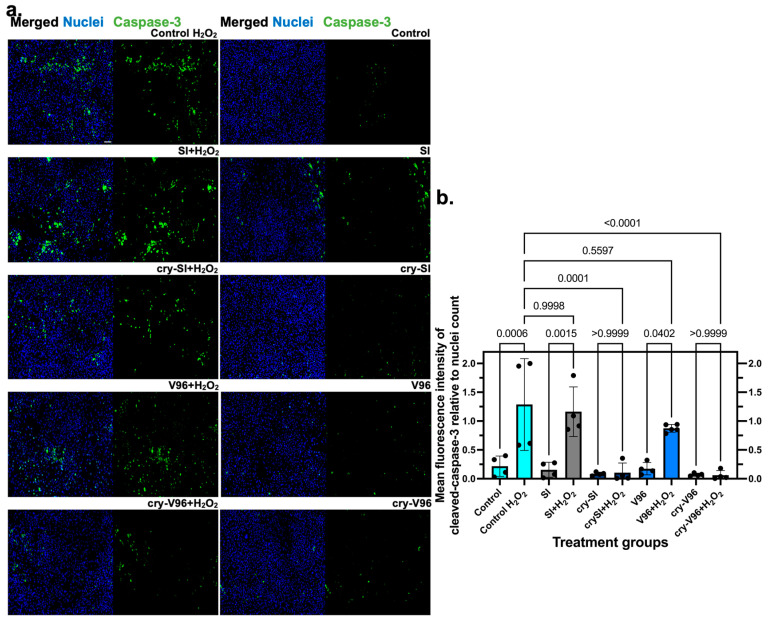
cry-ELPs suppress cleaved caspase-3 signal activation under oxidative stress. ARPE-19 cells were starved (1% FBS), pre-treated with 25 µM of SI, cry-SI, V96, and cry-V96, challenged on the next day by 200 µM of H_2_O_2_ overnight, fixed, and then stained by DAPI (blue) for further immunofluorescence detection of cleaved caspase-3. (**a**) Only cry-ELPs reduced activation of cleaved caspase-3 in ARPE-19 cells under oxidative stress. There is a noticeable decrease in fluorescent (green) apoptotic cells in the groups treated with cry-ELPs, compared to the positive control ones. (**b**) The data were analyzed by one-way ANOVA (*n* = 4–5, mean ± SD) and *p*-values are indicated next to post hoc comparisons. Only cry-SI and cry-V96 groups were protected against H_2_O_2_-induced cleaved caspase-3 activation. Scale bar = 100 µm.

H_2_O_2_-treated cells have been widely used as a model for oxidative stress to screen neuroprotective therapies [40]. Immunofluorescence-based assays are powerful methods for detecting, quantifying cellular proteins [41,42]. To validate this model, ARPE-19 cells were incubated with H_2_O_2_ to select a concentration (200 µM) with reproducible cell death that leaves the majority of cells attached for imaging (Supplementary Figures S1 and S2). Since cleaved caspase-3 is a crucial executioner of programmed cell death [43], it was then detected by immunofluorescence microscopy under normal and H_2_O_2_ challenge for all four constructs. Immunofluorescence epifluorescence microscopy was obtained through a 10× objective lens to visualize many cells (~800–2000 cells per field) (Figure 3a). In general, 25 µM ELPs have been fixed throughout the manuscript for both cry-SI and cry-V96, which leads to bulk phase separation at 26 and 30 °C, respectively. Thus, at physiological temperatures, they will coacervate. Differences between their activity should relate to their cellular activity, and not just their phase separation. Similar to controls, oxidative challenge with H_2_O_2_ enhanced cleaved caspase-3 activity for both V96 and SI alone (Figure 3b). In contrast, both cry-SI and cry-V96 completely blocked cleaved caspase-3 activation. Thus, both mini-cry ELP conjugates are similarly effective in protecting ARPE-19 against oxidative stress.

### 3.4. cry-ELPs Are Translocated to the Nucleus under Oxidative Stress

**Figure 4:**
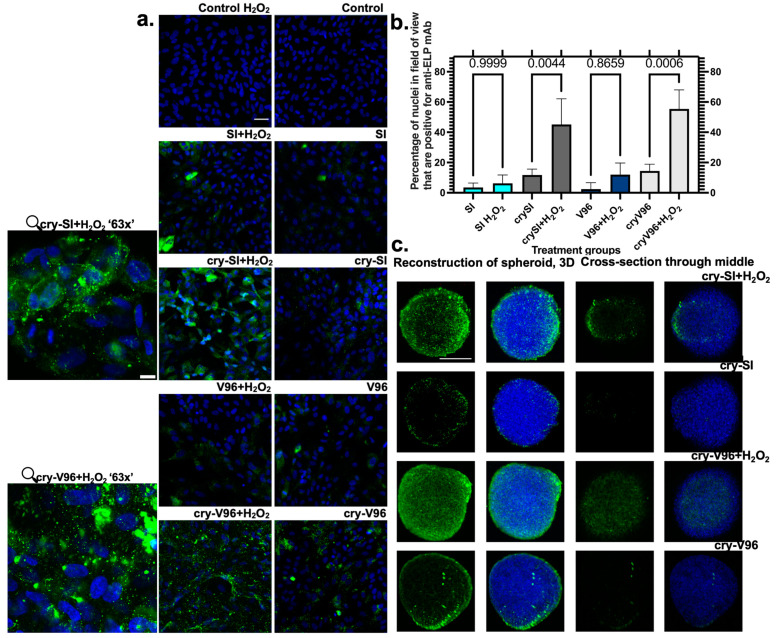
cry-ELPs penetrate 3D spheroids and associate with 2D cell culture under oxidative stress. ARPE-19 cells were pre-treated with 25 µM ELPs, exposed to oxidative challenge with 200 μM of H_2_O_2_, and an anti-ELP antibody (green) was used to detect ELP localization. Nuclei were counterstained by DAPI (blue). (**a**) Confocal microscopy imaging is shown using 20× lens ‘scale bar = 40 µm. Following H_2_O_2_ challenge, only cry-ELPs enhance cellular association, of which a green fraction becomes visible within the nucleus. The 63× magnification ‘scale bar = 10 μm’ image magnifies the intracellular localization of cry-ELPs, which in some cases overlap with the nucleus. (**b**) A total of ~60% of cells have staining of cry-SI and cry-V96 within the nucleus following oxidative challenge. ANOVA identified significant differences between groups, from which post hoc comparisons are indicated along with corresponding p-values. There was no difference between SI and V96 groups; however, both cry-SI and cry-V96 significantly enhanced nuclear staining upon H_2_O_2_ challenge. (**c**) To augment monolayer cell cultures, ARPE-19 spheroids were imaged by confocal microscopy. Spheroids are presented either as a 3D reconstruction of a z-stack or as an optical cross section through the middle of each spheroid ‘scale bar = 100 μm’. There is increased accumulation of the anti-ELP (green) signal for only cry-ELPs under oxidative challenge.

Many reports note the localization of αB-crystallin in the nucleus under oxidative stress; however, the mechanism remains unclear. Some mechanisms that may play a role include (i) the Hikeshi [44] protein that is a nuclear import carrier for the ATP form of Hsp70s; (ii) the Survival Motor Neuron (SMN) complex [45], which is a nuclear import carrier for the phosphorylated αB-crystallin; and (iii) the endosome–lysosome degradation pathway [46]. Mechanisms such as these might explain previously observed nuclear import of mini-cry ELPs, which is necessary for protection against stress [14]. Based on this, the nuclear localization of all peptides was monitored. A monolayer of ARPE-19 cells was treated with ELPs for one day, exposed to H_2_O_2_ for another day, and ELPs were detected by anti-ELPs immunofluorescence (Figure 4a,b). To further explore the cellular association and penetration, a three-dimensional (3D) spheroid of cells was used to evaluate cry-SI and cry-V96 under stress (Figure 4c). Spheroids are tools to explore the therapeutic interaction with a model closer to that of tissues in vivo [47]. When assessed using confocal microscopy, it was possible to visualize the entire 3D stack of images as well as a cross section through the spheroid. Both cry-SI and cry-V96 showed better nuclear accumulation, compared to V96 and SI. While the pattern of cellular staining and spheroid penetration differed slightly, cry-V96 more uniformly stains the surface and interior of H_2_O_2_-treated spheroids.

### 3.5. cry-ELPs Protect the ARPE-19 against Dextran Release

**Figure 5:**
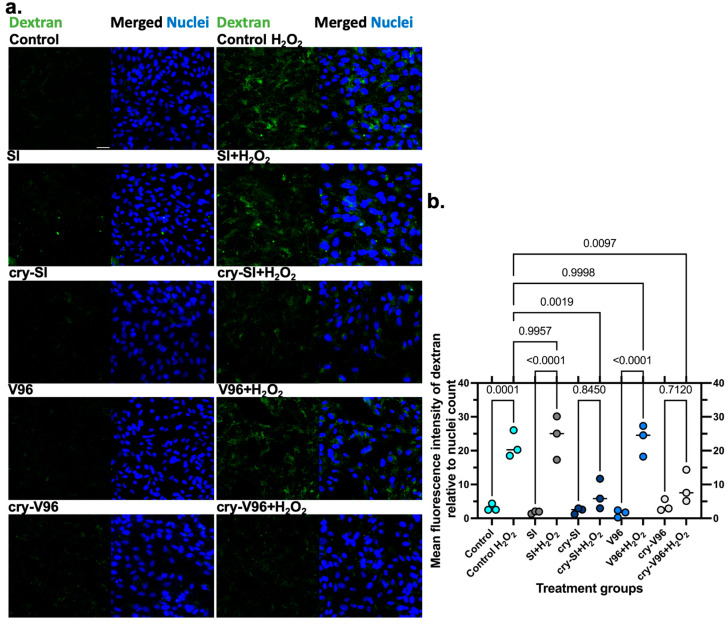
cry-ELPs reduce lysosomal permeabilization of dextran under oxidative challenge. ARPE-19 cells were pre-treated with 25 µM of ELPs for 24 h, incubated with 200 µM of H_2_O_2_ overnight, and 25 μM of dextran (green) was added to all groups. Cells were fixed and nuclei were stained by Hoechst (blue). (**a**) The results were collected by a 20× objective using confocal microscopy. In general, H_2_O_2_ challenge increased the signal from dextran; however, there was a noticeable decrease for cells treated with crystallin ELPs. (**b**) Images were quantified based on the mean green fluorescence intensity of dextran to the nuclei count. ANOVA identified significant differences between groups, from which significant post hoc comparisons are indicated along with corresponding *p*-values. As shown (*n* = 3, mean ± SD), control H_2_O_2_ ‘positive control’ and SI, V96-treated groups had the most dextran release under the H_2_O_2_ challenge, compared to their normal control. There was no statistical difference between SI + H_2_O_2_, V96 + H_2_O_2_, and the positive control. Similarly, cry-SI and cry-V96 exhibited no statistical difference in dextran release under oxidative stress, compared to the non-stressed condition. Most surprisingly, both cry-SI and cry-V96 significantly reduced the release of dextran compared to the positive control, which suggests the protective role provided by pre-treatment. Scale bar = 20 µm.

Lysosomal permeabilization, a sign of programmed cell death, is associated with cytotoxic stimuli, like H_2_O_2_ [48]. Being large proteins, cry-V96 and cry-SI appearance in the nucleus implies they have a mechanism to translocate from the endolysosomal pathway to the cytoplasm. A plausible mechanism is that lysosomal membrane permeabilization triggered by oxidative stress facilitates this translocation. To explore this possibility, dextran was used as an indicator of loss of endolysosomal integrity [49,50]. To do so, this study evaluates the protection of mini-cry ELPs from the lysosomal escape of 70 kDa FITC-Dextran. ARPE-19 were sequentially incubated with ELPs, challenged with H_2_O_2_, and finally incubated with 25 μM FITC-Dextran. After fixation, the uptake/release of dextran was observed using confocal microscopy (Figure 5a). Dextran was clearly released more following H_2_O_2_ stress, with the exception of the cry-ELPs (Figure 5b). These results possibly correlate with reports that implicate αB-crystallin in modulating lysosome function [51,52]. Consistent with (Figure 2 and Figure 3), these results demonstrate the protective potential of cry-SI and cry-V96 against lysosomal membrane permeabilization.

### 3.6. cry-ELP Colocalization with Lysosomes Decreases under Oxidative Stress

**Figure 6:**
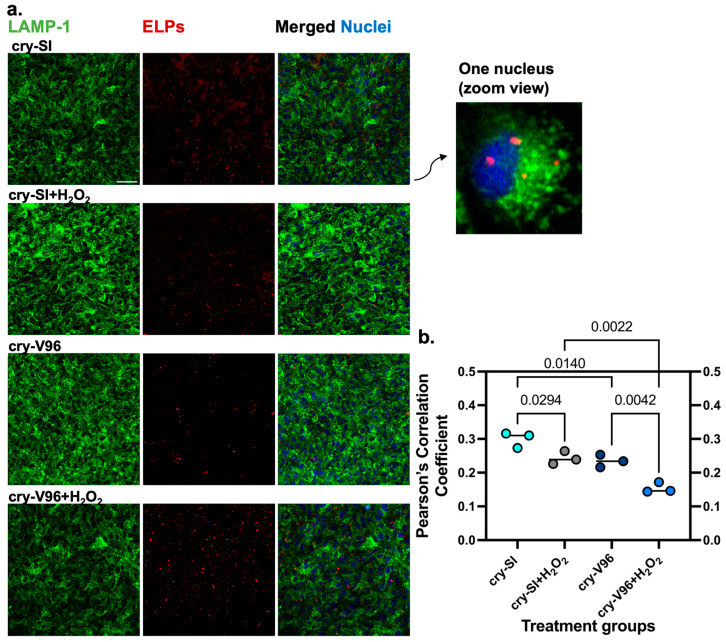
Oxidative stress reduces cry-ELPs colocalization with the lysosomal compartment, LAMP-1. ARPE-19 cells were treated with both 25 µM of rhodamine-labeled ELPs (red) and 200 µM of H_2_O_2_ for 1 h and compared with unchallenged cells using confocal microscopy. (**a**) Secondary immunofluorescence against LAMP-1 (green) was used to label lysosomes and observe colocalization with ELPs. Nuclei were stained by Hoechst (blue). (**b**) Colocalization between ELPs and LAMP-1 was used to quantify escape in the images (*n* = 3, mean ± SD). ANOVA identified significant differences between groups, from which significant post hoc comparisons are indicated along with corresponding *p*-values. For both cry-SI and cry-V96, H_2_O_2_ challenge significantly reduced colocalization between the ELPs and lysosomes, which is consistent with lysosomal permeabilization. Interestingly, cry-V96 has lower colocalization compared to cry-SI under both stressed and non-stressed conditions. This suggests that cry-V96 has a greater capacity to escape lysosomal trafficking, which may enhance its potential efficacy. Scale bar = 50 µm.

Having demonstrated that H_2_O_2_ challenge promotes appearance of cry-ELPs in the nucleus (Figure 4) and that they protect against lysosomal permeabilization by dextran (Figure 5), colocalization of cry-ELPs with lysosomes (LAMP-1) was assessed (Figure 6a). A likely step on pathway of cry-ELP uptake, the LAMP-1 cellular compartment is typically associated with late endosome and lysosomal membranes [53,54]. To assess this, cells were co-incubated with rhodamine-labeled cry-ELPs and H_2_O_2_ for 1 h. Cells were fixed, permeabilized, and stained for LAMP-1 by secondary immunofluorescence. Cells were imaged by confocal microscopy and quantified by PCC analysis (Figure 6b). In general, cry-ELPs overlapped with LAMP-1. H_2_O_2_ challenge reduced the colocalization of both cry-ELPs with LAMP-1, which is consistent with endolysosomal escape (Figure 4). cry-V96 had the lowest colocalization with lysosomes, which suggests it either avoids lysosomal trafficking and/or has greater endolysosomal escape.

### 3.7. cry-ELPs Colocalize with Dextran

**Figure 7:**
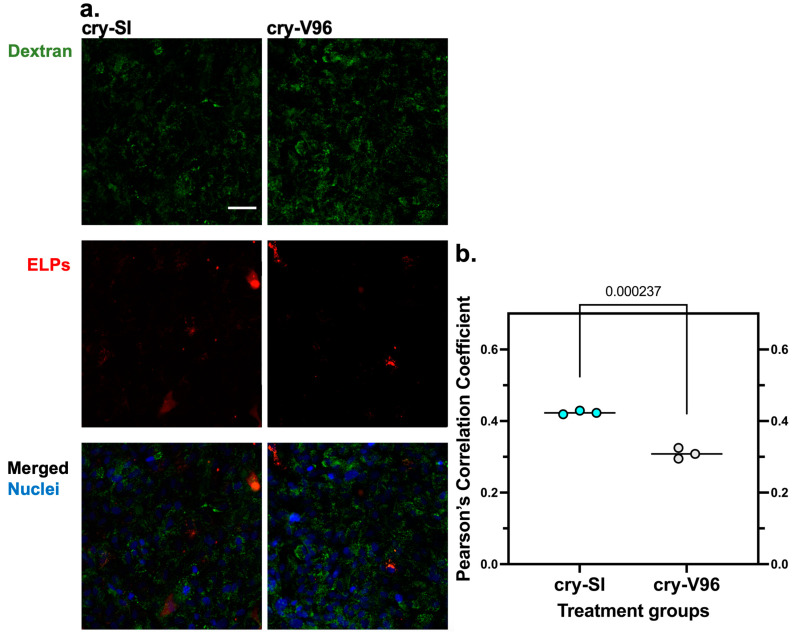
cry-ELPs colocalize with dextran. ARPE-19 cells were pre-incubated with 25 µM of rhodamine-cry-ELPs (red) for 24 h, washed, and 25 µM of dextran (green) was applied to both groups. (**a**) Nuclei were stained by Hoechst (blue), images were visualized by confocal microscopy ‘20× lens’ and analyzed based on the pixel-by-pixel covariance. (**b**) Colocalization between cry-SI and cry-V96 and dextran were quantified using PCC. The statistical significance was compared by Student’s *t* test (*n* = 3, mean ± SD). cry-V96-treated cells had slightly lower PCC values, suggesting less colocalization with dextran than cry-SI. Scale bar = 50 µm.

Since cry-ELPs colocalize with lysosomes (Figure 6) and protect against lysosomal membrane permeabilization of dextran (Figure 5), this raised the possibility that dextran and cry-ELPs follow a similar pathway of cellular uptake. To explore this, 70 kDa dextran was used as a fluid-phase endocytic marker and evaluated for colocalization with cry-ELP. ARPE-19 were treated sequentially with 25 µM of ELPs overnight, then incubated with 25 µM of FITC-Dextran for 24 h, and fixed for microscopic evaluation (Figure 7a). The settings were also unified for the comparative groups. Similar to above (Figure 6), PCC results indicated significantly greater colocalization of cry-SI compared to cry-V96 (Figure 7b).

## 4. Discussion

This report compares the mechanism of protection for two related formulations of the mini-cry peptide from the αB-crystallin protein. Many studies have confirmed the interplay between chaperone and defensive role of α-crystallins in ocular disorders [55,56,57]. Kannan and coworkers [55] reported that the absence of α-crystallin exacerbated retinal degeneration in a hypoxic in vivo model, suggesting a protective role of α-crystallin in preventing retinal degeneration under stress. Munemasa and coworkers [56] demonstrated that the expression of αA and αB-crystallins was associated with increased survival of a retinal ganglion degeneration in vivo model by 95% and 75%, respectively. Recently, Hazra and coworkers [57] showed αB mini-peptides decreased inflammatory cytokines and enhanced cellular recovery following lamellar flap surgery. Correspondingly, DLS data in this report confirmed chaperone activity of mini-cry conjugated to two different ELPs (Figure 2), compared to controls. Moreover, the immunofluorescence detection of cleaved caspase-3 revealed that pre-treated cells with mini-cry ELPs had the lowest cleaved caspase-3 signal and the most protection against 200 µM H_2_O_2_ (Figure 3). This 200 μM concentration was selected as the challenge because it promoted 18.9 ± 2.7% of the cells to enter apoptosis, while retaining 85.6 ± 2.7% viability (Figures S1 and S2). Higher concentrations above 800 μM dramatically reduce viability, which is unlikely to be apoptosis. Some studies [20,58] highlight the importance of avoiding lethal doses of H_2_O_2_ that induce irreversible cell death, which confounds interpretation. The sensitivity of ocular tissues to oxidants remains a challenge to model in a way that is relevant to other pathophysiological conditions [59,60]. Haendeler and coworkers [61] proved that short exposure of 10 and 50 μM of H_2_O_2_ was sufficient to alter Trx-1 mRNA levels, the redox regulator thioredoxin-1, which affected cell growth and apoptosis. Also, Song and coworkers [62] demonstrated a 200 μM challenge of H_2_O_2_ correlated with p62 elevation, a multifunctional scaffold protein with various cell signaling associations, for both primary RPE or ARPE-19 cells. While an ISO 10993-5 standard suggests that apoptosis should be induced up to 30% for cellular toxicity assessments, 200 μM H_2_O_2_ selected in this study induced reliable, statistically significant levels of apoptosis. As mentioned above, this concentration is consistent with prior studies of RPE cell cultures. Future studies should certainly evaluate the upper concentrations of H_2_O_2_ challenge that can be reversed by crystallin peptides. Furthermore, crystallins are distinguished by their cellular import following cellular stress. Despite published reports on the internalization of crystallin peptides, a comprehensive mechanism of endolysosomal escape and nuclear translocation is not fully elucidated. den Engelsman and coworkers [45] proposed that αB-crystallin undergoes serine phosphorylation, which allows C-terminal interaction with gemin3. An integral part of the SMN complex, this interaction is thought to promote nuclear import. As evidence, that group replaced serine residues at positions 19th, 45th, and 59th with negatively charged aspartate residues and investigated nuclear entry. These mutations hindered transport of αB-crystallin into the nucleus. Furthermore, they showed knockdown of gemin3 resulted in cytoplasmic accumulation of αB-crystallin. In another report, Kose and coworkers [44] identified that the ATP form of crystallin contributes to its nuclear import during cellular stress. Transport was associated with the Hikeshi carrier, an ATPase cycle-driven carrier. This complex translocates through nuclear pores via interaction with phenylalanine-glycine (FG) repeat-containing nucleoporins. Van Rijk and coworkers suggest the nuclear localization of αB-crystallin depends on the splicing factor, SC35 [63]. It was proposed that nuclear import is an essential step for downregulating nuclear-apoptotic mechanisms and thereby, controlling cellular fate [64]. Indeed, our 2D and 3D culture results also confirmed the cellular association and deep penetration of crystallin-conjugated formulations under oxidative stress (Figure 4).

While two reports indicate intracellular delivery of crystallin peptides using cell-penetrating peptides (CPPs) [65,66], ELP conjugation can extend pharmacokinetics and prolong therapeutic activity between injections. Intravitreal injection is a clinically accepted, relatively safe procedure to treat ocular disorders. Since it is performed intermittently under the care of a clinician, it promotes patient compliance and therapeutic efficacy [67]. Furthermore, ELP systems undergo physiological degradation by endopeptidases, making them a logical candidate for clinical delivery of peptide cargo, such as mini-cry [68]. Several years ago, our team first fused αB-crystallin onto two ELP motifs (VPGSG)_96_ and (VPGSG)_48_(VPGIG)_48_. Using (VPGSG)_96_, which has a hydrophilic guest residue, serine, led to a T_t_ of around 55 °C. When adding a hydrophobic (VPGIG)_48_ block, the resulting cry-SI formed an inverted micellar system with a multi-step phase separation (Figure 1c) and a T_t_ of 30 °C. Upon intravitreal injection, it resulted in a mean residence time in the retina of ~3 days [31]. As such, cry-SI is a promising platform for extended release upon injection at physiological temperature. To explore the effect of ELP architecture on intracellular delivery, this manuscript now compares the cry-SI diblock with cry-V96, which uses (VPGVG)_96_ as a hydrophobic block. This fusion, cry-V96, similarly phase separates around 37 °C, but shows a single-step phase separation (Table 1, Figure 1c), which may alter ocular retention. It is unknown how the change from diblock SI to monoblock V96 ELP might affect its protective potential; however, this report now shows that cry-V96 is at least equivalent to cry-SI. Future studies will compare their pharmacokinetic and therapeutic behavior in the retina.

When cry-ELPs are added to cells, they become internalized by endocytosis-mediated mechanisms, as previously reported by our group [14]. It was seen that their uptake was driven by clathrin and dynamin pathways. Upon inhibiting these pathways, both nuclear localization and protective roles were abolished. To elaborate on this mechanism, here we tracked LAMP-1, a lysosomal marker to investigate formulation fate following the endocytosis. As observed (Figure 6), colocalization with LAMP-1 was significant, but also decreased significantly upon oxidative stress. ELPs typically require additional mechanisms to escape endolysosomal degradation. For example, Massodi and coworkers engineered a CPP-functionalized ELP and explored it for cancer therapy [69]. Interestingly, they concluded that the ELP conjugate had more potent cancer cell death than the CPP control, suggesting better intracellular interaction and delivery. In another relevant study from Yeboah and coworkers [70], they compared the therapeutic outcome of SDF1, a stromal cell-derived growth factor-1, to an SDF1-ELP conjugate. Compared to free SDF1, SDF1-ELP conjugate exhibited faster biological activity. Herein (Figure 6b), the results also revealed that cry-V96 had the greatest ability to reduce colocalization with lysosomes, which may indicate lysosomal escape. de Vrueh reported that conjugation of L-valine to acyclovir enhanced transport of acyclovir in a Caco-2 cell line by 7-fold [71]. More recently, Jain and coworkers concluded that L-valine-decorated nanoparticles enhanced oral delivery of insulin, suggesting selective binding to oligopeptide-mediated transporters [72]. Other related studies have confirmed the role of valine in promoting cellular uptake, permeability, and therapeutic benefit [73,74,75]. In addition to the possible role of valine in potentiating therapeutic delivery, αA or αB-crystallin uptake was dependent on sodium-coupled oligopeptide transporters 1 and 2 (SOPT1, SOPT2). Sreekumar and coworkers [76] used DADLE and deltrophin II as competitive substrates to SOPT1 and SOPT2 to investigate the preferential uptake of these transporters. Intriguingly, α-crystallins showed dose-dependent inhibition of these substrates, suggesting affinity-related mechanisms. It is not only valine-mediated constructs that may promote internalization, but also micellar platforms such as SI [77]. For example, the Chilkoti group [78] demonstrated that integrin-targeting polypeptide micelle has a 1000-fold increase in avidity to αvβ3 receptor, compared to monomer ligand. Possibly, the conjugation of mini-cry to SI led to an inverted micelle and impacted the cellular entry, compared to cry-V96. Also, the dextran colocalization analysis (Figure 7b) revealed that the cry-V96-treated group was less colocalized with dextran, compared to the cry-SI group. This suggests cry-V96 may have a greater capacity to avoid the endolysosomal pathway used by dextran than cry-SI.

It is known that oxidative stress and the resultant radicals lead to functional impairments to organelles, including lysosomes [79]. As shown by cleaved caspase-3 activity, internalization culture studies (Figure 3 and Figure 4), 24 h are enough for allowing the cellular machinery to export the cry-ELPs into the nucleus and accordingly exhibit a protective function. It is also not surprising that the dextran permeabilization assay (Figure 5) revealed that cry-ELP-treated groups release less dextran under oxidative stress. Wang and coworkers [51] demonstrated that αB-crystallin-pre-treated cells had a notable effect on decreasing the number of autophagolysosomes in LPS-stimulated cells, indicating a prophylactic beneficial effect. Other studies suggest an interactive role of crystallin peptides in lysosomal acidification and recycling [52,80,81]. As shown in (Figure 5), SI- and V96-treated groups under oxidative stress had more lysosomal release, while lysosomes in the cry-ELP-treated groups showed no significant dextran release under stressed and non-stressed conditions, suggesting that their lysosomes remain intact.

Other therapeutic opportunities can benefit from lysosomal compartmentalization of ELPs [82]. ELP technology can be manipulated to directly interfere with oxidative stress through its endocytic pathway and even evade lysosomal degradation [48]. Herein, as shown in (Figure 6), the lysosomal release under H_2_O_2_ challenge is an intriguing means to promote ELP release for further interaction with cytosolic and nuclear targets (Figure 3 and Figure 4).

## 5. Conclusions

This manuscript compares two successful ELP conjugates for intracellular delivery of a mini chaperone peptide from αB-crystallin. A new construct, cry-V96, was compared to our previously published cry-SI. According to 2D and 3D ARPE-19 culture models, both constructs show notable effects, while cry-V96 appears to have slightly better cellular penetration, higher lysosomal escape, and lower colocalization with dextran. A limitation of this study is our selection of a single concentration of H_2_O_2_ to induce acute toxicity. Future studies should evaluate the protective response across a wider range of oxidative stressors, including at different concentrations and durations of exposure. Having demonstrated that both constructs have similar levels of chaperone activity and protection against H_2_O_2_-induced cell death, both remain candidates for future comparative studies of intraocular pharmacokinetics and therapeutic efficacy using different models of oxidative stress in the retina. In light of the significant interaction between the uptake of crystallin ELPs and oxidative challenge, the next steps will be to evaluate a broader range of oxidants and their effect on endolysosomal trafficking, cytoplasmic release, nuclear accumulation, and effects on mitochondrial function.

## Figures and Tables

**Table 1:** Identification of biophysical properties of ELPs evaluated in this study.

Peptide Nomenclature	Amino Acid Sequence ^a^	Expected MW ^b^ (kDa)	Observed MW ^c^ (kDa)	T_t_, Intercept ^d^, *b*, (°C)	T_t_, Slope ^e^, *m*, (°C/Log (μM))
SI 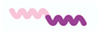	MG(VPGSG)_48_(VPGIG)_48_Y	39.6	39.7	77.3	4.2
				[69.9 to 84.7]	[1.5 to 9.9]
cry-SI 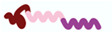	MGDRFSVNLDVKHFSPEELKVK G(VPGSG)_48_(VPGIG)_48_Y	42.1	42.0	30.1	3.4
				[26.7 to 33.5]	[0.03 to 6.8]
V96 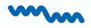	MG(VPGVG)_96_Y	39.6	39.6	36.7	4.0
				[35.7 to 37.7]	[3.0 to 5.0]
cry-V96 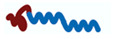	MGDRFSVNLDVKHFSPEELKVK G(VPGVG)_96_Y	42.0	42.0	35.2	4.3
				[34.5 to 36]	[3.5 to 5.0]

^a^ Mini-cry amino acid sequence (20-mer) is as follows; DRFSVNLDVKHFSPEELKVK. ^b^ Determined by SnapeGene using open reading frame, excluding the Methionine ‘starting codon’. ^c^ Determined by MALDI-TOF [M+H]^+^. ^d,e^ Determined by fitting OD_350_ phase behavior using Equation (4) to relate T_t_ and concentration. (95% confidence interval).

## Data Availability

Data used to prepare this study are available through contact with the corresponding author.

## Supplementary Materials

The following supporting information can be downloaded at: https://www.mdpi.com/article/10.3390/antiox12101817/s1.
