# Supplementary material for: αB-Crystallin Peptide Fused with Elastin-like Polypeptide: Intracellular Activity in Retinal Pigment Epithelial Cells Challenged with Oxidative Stress
Source: Antioxidants (Basel). 2023 Sep 30;12(10):1817. doi: 10.3390/antiox12101817 (PMC10604459; doi:10.3390/antiox12101817)
Supplement: Supplementary file 1 [file antioxidants-12-01817-s001.zip › antioxidants-2610200-supplementary.pdf]

αB-crystallin peptide fused with elastin-like polypeptide: intracellular activity in retinal pigment epithelial cells challenged with oxidative stress

Sara Aly Attia[1], Anh Tan Truong[1], Alvin Phan[1], Shin-Jae Lee[2], Manal Abanmai[1,3], Marinella Markanovic[1], Hugo Avila[1], Haozhong Luo[1], Atham Ali[1], Parameswaran G Sreekumar[4], Ram Kannan[4,5], J. Andrew MacKay[1,2,6*].

1 Department of Pharmacology and Pharmaceutical Sciences, USC Mann School of Pharmacy and Pharmaceutical Sciences, University of Southern California, USA.
2 USC Mann Department of Biomedical Engineering, Viterbi School of Engineering, University of Southern California, USA.
3 Department of Pharmaceutics, College of Pharmacy, Prince Sattam Bin Abdulaziz University, Saudi Arabia.
4 Doheny Eye Institute, USA.
5 Stein Eye Institute Geffen School of Medicine, University of California, USA.
6 Department of Ophthalmology, Keck School of Medicine, University of Southern California, USA.
* Authors to whom correspondence should be addressed: jamackay@usc.edu.

This work is dedicated to the memory of our beloved collaborator David R Hinton, MD, FARVO.

Citation: To be added by editorial staff during production.

Academic Editor: Firstname Lastname

Received: date
Revised: date
Accepted: date
Published: date

Copyright: © 2023 by the authors. Submitted for possible open access publication under the terms and conditions of the Creative Commons Attribution (CC BY) license (https://creativecommons.org/licenses/by/4.0/).

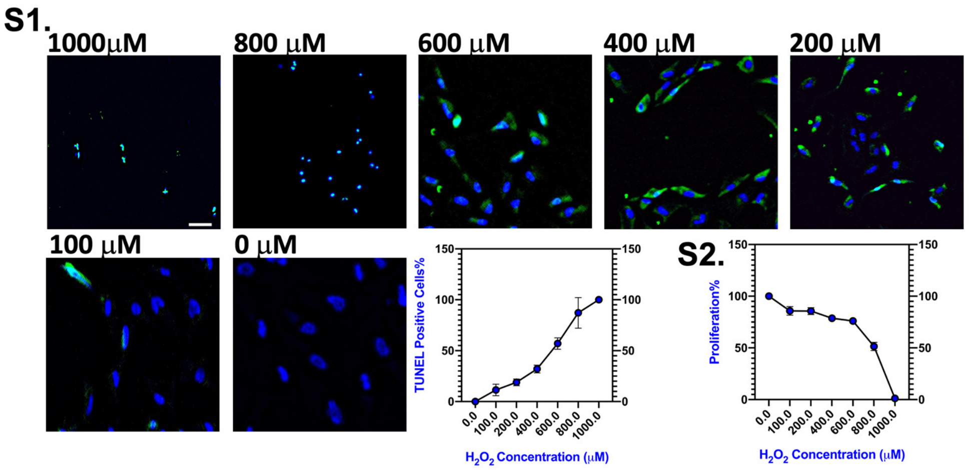

Figure S1,2. ARPE-19 cells exhibit H_2O_2 dose-dependent induction of apoptosis and inhibition of viability. S1) Cells were starved in 1% FBS during treatment by the following concentrations: 1000, 800, 600, 400, 200, and 100 μM of H_2O_2. The TUNEL assay was used to detect the apoptotic (nucleus positive) cells (green) as a percentage of total nuclei stained with DAPI (blue). Images were obtained using a Keyence epifluorescence microscope. S2) ARPE-19 cells were treated by the same concentrations of H_2O_2 but were instead investigated for cellular viability using the formazan-based WST-1 assay. 200 μM H_2O_2 challenge was selected for further experimental studies in the manuscript because that concentration induces 18.9 ± 2.7% of the cells to enter apoptosis, while retaining 85.6 ± 2.7% of cell viability (n = 3, mean ± SD) using the TUNEL, WST-1 results, respectively. (Scale bar = 200 μm)
